# Tunable Oxidized-Chitin Hydrogels with Customizable Mechanical Properties by Metal or Hydrogen Ion Exposure

**DOI:** 10.3390/md22040164

**Published:** 2024-04-03

**Authors:** Angelica Mucaria, Demetra Giuri, Claudia Tomasini, Giuseppe Falini, Devis Montroni

**Affiliations:** Department of Chemistry “Giacomo Ciamician”, University of Bologna, Via F. Selmi 2, 40126 Bologna, Italy; angelica.mucaria2@unibo.it (A.M.); demetra.giuri2@unibo.it (D.G.); claudia.tomasini@unibo.it (C.T.); giuseppe.falini@unibo.it (G.F.)

**Keywords:** chitin, gel, oxidation, calcium, pH

## Abstract

This study focuses on the optimization of chitin oxidation in C6 to carboxylic acid and its use to obtain a hydrogel with tunable resistance. After the optimization, water-soluble crystalline β-chitin fibrils (β-chitOx) with a degree of functionalization of 10% were obtained. Diverse reaction conditions were also tested for α-chitin, which showed a lower reactivity and a slower reaction kinetic. After that, a set of hydrogels was synthesized from β-chitOx 1 wt.% at pH 9, inducing the gelation by sonication. These hydrogels were exposed to different environments, such as different amounts of Ca^2+^, Na^+^ or Mg^2+^ solutions, buffered environments such as pH 9, PBS, pH 5, and pH 1, and pure water. These hydrogels were characterized using rheology, XRPD, SEM, and FT-IR. The notable feature of these hydrogels is their ability to be strengthened through cation chelation, being metal cations or hydrogen ions, with a five- to tenfold increase in their storage modulus (G’). The ions were theorized to alter the hydrogen-bonding network of the polymer and intercalate in chitin’s crystal structure along the a-axis. On the other hand, the hydrogel dissolved at pH 9 and pure water. These bio-based tunable hydrogels represent an intriguing material suitable for biomedical applications.

## 1. Introduction

Hydrogels are materials characterized by a crosslinked 3D network of polymeric molecules, fibers, or particles, in which the aqueous phase acts as a dispersing medium. Due to their nature, these materials have the ability to absorb and retain large quantities of water without dissolving or undergoing significant changes in shape [1,2].

Hydrogels can be classified based on their framework molecules, method of synthesis, and the type of interactions (i.e., chemical or physical) that define them. However, a relevant distinction is made between synthetic and natural hydrogels, depending on the source of the constituent molecule or molecules [3]. The latter are frequently preferred over their synthetic counterparts due to their reproducibility, biodegradability, non-toxicity, and biocompatibility. Currently, a variety of natural biopolymers are used to prepare hydrogels including cellulose, hemicellulose, chitin, chitosan, gelatin, agarose, starch, hyaluronic acid, alginate, collagen, and DNA [2,4]. Among these biopolymers, chitin has attracted great interest due to its huge reserves in nature, being the second most abundant biopolymer on Earth [5,6], high hydration, and strong network of hydrogen bonds [7]. All these properties make it a promising candidate for the preparation of highly swelling hydrogel systems [8,9]. Chitin exists in nature as three polymorphs, α-chitin, β-chitin, and ɣ-chitin, which differ in their arrangements of polymeric chains: anti-parallel, parallel, and alternating, respectively [10]. The α-chitin is the most abundant polymorph and can be found in arthropods cuticles, fungi cell walls, or the cysts of *Entamoeba* [11,12]. The β-chitin is still abundant in nature and can be found in mollusks, or other biomineralizing organisms, as *Foraminifera* [13,14]. Finally, the ɣ-chitin is extremely rare and its actual existence is still debatable. To date, this last polymorph has been documented in the cocoon fibers of the *Orgya dubia*, the stomach of *Loligo*, and some insects’ peritrophic membranes [5,15].

The α-chitin is the most crystalline polymorph, with a crystallinity generally greater than 80% [16]. It is characterized by a strong network of hydrogen bonds that lead to a high mechanical modulus. On the other hand, the β-chitin has a lower crystallinity and fewer intermolecular forces. This has led to a special interest in this polymorph since it is more prone to functionalization and solubilization compared to α-chitin [15].

Chitin-based hydrogels have shown promising application prospects in the field of biomedicine [2]. The usual method for gelling native chitin involves cross-linking chitin molecules in solutions using physical or chemical methods [17]. However, the main difficulty in making chitin hydrogels is the lack of appropriate solvents due to chitin’s insolubility. Several methods have been proposed to address this issue, including the use of specialized solvents to dissolve native chitin [18,19,20,21,22,23], hydrophilic modification [24,25], and the defibrillation of the material [1].

In Fan et al. (2008), a series of LiOH/KOH/urea aqueous solutions with different weight ratios were used to dissolve chitin structures with degrees of acetylation ranging from 5% to 94%. Then, coagulants were applied to these chitin/chitosan solutions to form robust hydrogels [16]. In Dang et al. (2020), the solubilization of chitin was carried out in a CaCl_2_ methanol solution. Chitin hydrogels were then formed by adding excess water to the chitin solution and removing the methanol and calcium ions through dialysis or filtration [19]. In all these studies, organic solvents or other potentially toxic chemicals were used to achieve chitin solubilization.

Chitosan, an acid-soluble deacetylated derivative of chitin, allows hydrogels to be obtained by exploiting the interactions of the cationic amino groups of chitosan, with negatively charged molecules and anions. Chitosan can form an ionic complexation with small anionic molecules, such as sulfates, citrates, and phosphates [26,27], or metal anions such as MoO_4_^2−^ [28,29].

In contrast, in Ma, Qinyan, et al. (2019), a β-chitin fiber solution was obtained using ammonium persulfate as an oxidizing agent to introduce a carboxylic group at the C6 position of chitin [30].

Other oxidation methods are also reported in the literature. 2,2,6,6-Tetramethylpiperidine 1-oxyl (TEMPO) is often used as a primary oxidizing agent in combination with other co-oxidants, as in the system TEMPO/NaClO/NaBr [31,32]. However, the use of TEMPO to obtain soluble fibrils is a combined process often involving other steps such as mechanical disintegration, sonication, or the enzymatic, or acid hydrolysis method [33].

Oxidation with periodate is also reported. The reaction involves pre-treating chitin with KOH for 24 h and using periodic acid to ensure the correct amount of periodate ions in the reaction solution. The reaction requires long reaction times, such as 14 days, as reported by Liu et al. (2021) [34].

The carboxylic acid introduced with these methods facilitates the defibrillation of the system by creating negative charges that repel each other in a basic environment.

We chose to follow the oxidation method with ammonium persulfate as reported in Ma, Qinyan, et al. (2019), since our work represents a continuation of theirs. Additionally, the use of ammonium persulfate allows for chitin fibers to be obtained with fewer reaction steps and less time required, compared to other oxidants used.

The oxidized chitin fibers obtained by Ma, Qinyan, et al. (2019) were able to form hydrogels upon sonication. Following this method, the hydrogels could also potentially be formed by physical interactions (anion–cation and/or hydrogen bonding), avoiding the use of potentially toxic cross-linkers [35]. This potential was not explored in the 2019 paper.

Among biopolymers, oxidized chitin shows a similarity with alginate, as both structures have the C6 oxidized to a carboxylic group. However, in oxidized chitin, the degree of C6 oxidation is controlled and not complete as in alginate, which means the contribution of hydrogen bonding and apolar interactions is prevalent during gelation [36]. Moreover, alginate is solubilized as single molecules, while oxidized chitin dissolves/disperses as crystalline nano-fibrils. This difference affects the gelling capability, even if its mechanism is, in part, based on analogue interactions. Moreover, the presence of crystalline fibrils is expected to positively affect the mechanical resistance of the gel.

In this study, we aim to explore the potential preparation of hydrogels using both β-chitin and α-chitin as the starting material, aiming to explore their different reactivity. In Ma, Qinyan, et al. (2019), only β-chitin was studied. In addition to their work, our aim is to optimize their synthesis by controlling the starting material grain size and the oxidation conditions. By doing so, we expect to oxidize preferentially the chitin’s low crystalline regions, leaving the core of the crystalline motifs almost unaltered. This would allow us to obtain hydrogel building blocks with a higher value. Finally, we want to explore the tunability of the hydrogel’s mechanical properties by exposing it to diverse environments, as in the presence of metal ions or variable pHs.

These optimized hydrogels could lead to potential applications in diverse fields, including the biomedical one where a material’s adaptation to a changing environment could be crucial.

## 2. Results and Discussion

### 2.1. Chitin Grain Size Optimization

The first step of the work was to optimize the material synthesis, to obtain soluble crystalline fibrils of oxidized chitin. To maximize the yield of production and fasten the kinetic of oxidation, we decided to work with chitin powder, rather than flakes, to have a higher initial exposed surface. While α-chitin was already purchased as a powder, the purchased β-chitin was milled to obtain a fine powder. Both powders were sieved to obtain diverse grain size: (i) from 600 µm to 150 µm, (ii) from 150 µm to 45 µm, and (iii) below 45 µm.

A study on the crystallinity and structure of α-chitin and β-chitin powders with diverse grain sizes was carried out. The aim was to reduce the grain size of the powder while preserving the crystallinity as much as possible. Table 1 reports the diffraction angles and the crystallite size (d) of the main reflections obtained from the analyses of X-ray powder diffraction (XRPD) patterns of α-chitin and β-chitin powders sieved at different grain sizes. The diffractograms are reported in Figure S1. Miller indices were attributed according to literature data [37,38]. Chitin’s diffraction peaks are a convolution of different signals, and the Miller indices reported refers only to the most intense one, which contributes to most of the signal intensity.

In the α-chitin powders, a shift of the (020) and (110) reflections towards lower diffraction angles was observed when reducing the grain size dimension from 600 μm down to below 45 μm. This indicates an increase in the unit cell parameters, suggesting a weakening of interchain interactions. This process was also associated with a mild decrease in crystallinity in the sample with a grain size < 45 µm, as indicated by the decrease in the crystal size along the zone axis associated to the (110) plane and the decrease in the CI calculated on both planes.

The β-chitin powder samples showed lower crystallinity than α-chitin, when compared in the same grain size ranges. Similarly, to that observed for α-chitin, a progressive reduction in the crystallinity occurred also for β-chitin. In this case, the decrease was observed both along the [010] and [100] zone axes. Moreover, for this polymorph, an increase in the lattice cell parameters was revealed by the shift of the diffraction peaks to lower angles which, as for α-chitin, can be associated with a weakening of the interplane and interchain interactions.

From the XRD analysis, for the β polymorph, a decrease in crystallite size corresponding to the (100) reflection is observed as the particle size decreases. An overall decrease in the material crystallinity was observed from the CI of both peaks decreasing the grain size.

A decrease in crystallinity decreasing particle size was also reported in Jorge A. et al. (2018) [39]. Both α-chitin and β-chitin were ground in a knife mill and sieved according to the particle sizes.

However, for the α polymorph, less significant variations are observed. This is consistent with the different crystalline packing of the two polymorphs, more compact for α-chitin [39].

Based on the obtained results, samples of α-chitin and β-chitin powders having grain sizes between 600 μm and 150 μm were used for further experiments, being the ones retaining the highest crystallinity.

### 2.2. Optimization of the Oxidation Time for β-Chitin

The selected chitin samples, with a powder grain size of 600–150 μm, were oxidized by reacting with ammonium persulfate at a temperature of 40 °C following the procedure described in Section 3.4. This synthetic step was optimized in the reaction time.

**Figure 1:**
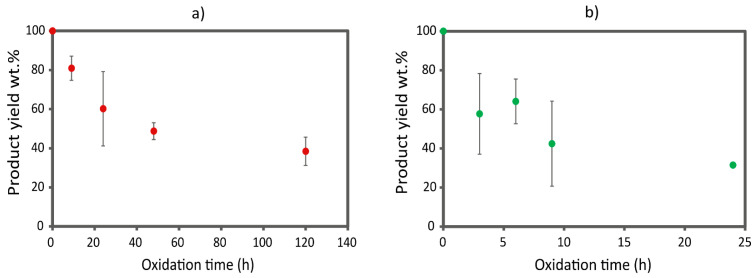
Product yield expressed as weight percentage obtained at the various oxidation times of (**a**) α-chitin and (**b**) β-chitin.

An initial evaluation of the reaction progress was monitored through turbidity measurements, as the heterogeneous reaction proceeds with the dissolution of chitin particles. After just 3 h of reaction, the initial grains in the β-chitin powder reduced their dimensions, as evidenced by the rapid decrease in transmittance of the reaction batch (Figure S2). After 24 h, the reaction mixture showed a complete dissolution of chitin, while, after 45 h, an increment in turbidity was observed due to aggregate formation. In parallel, the yield of the reaction decreased progressively while increasing the reaction time; see Figure 1. This can be attributed to the formation of chitin fibers of nanometric sizes that were lost during the purification of the product or the degradation of chitin due to excessive oxidation. At 45 h, it was no longer possible to collect material, even by dialysis, suggesting a complete degradation of the polysaccharide chain. Considering the turbidity profile obtained and the yield associated with that, we decided to focus our attention on the following reaction times, 3 h, 6 h, 9 h, and 24 h, to sample along different positions of the turbidity profile. No time over 24 h was examined since the yield was considered too low.

The obtained oxidized chitin powders were examined using solid-state nuclear magnetic resonance (ss-NMR), Fourier-transform infrared spectroscopy (FT-IR), and XRPD.

The FT-IR spectra (see Figure S3b) showed all the absorption bands of chitin, plus a new weak absorption band at 1730 cm^−1^. This band has been documented as a stretching of the C=O of carboxylic acids [30], demonstrating a positive functionalization of chitin. A ratio between this band and the band at 1073 cm^−1^ (a C–O stretching associated to the sugar ring) was initially used to monitor the oxidation along the time; the results are reported in Figure S3c.

**Figure 2:**
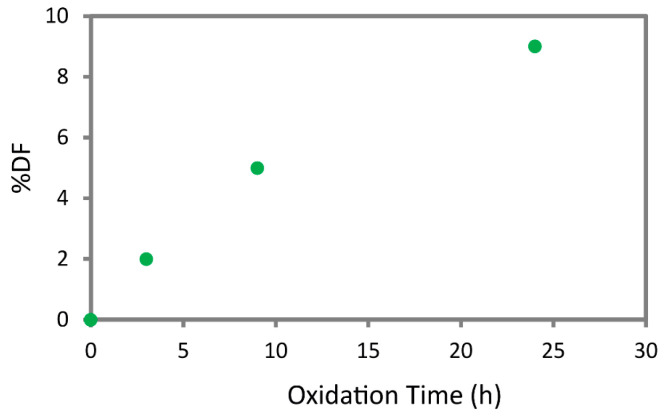
Degree of functionalization percentage (%DF) of C6 of β-chitin at different reaction times.

The ss-NMR spectra (see Figure S4) were used to calculate the degree of functionalization (%DF) of chitin. The %DF are reported in Figure 2 and show an incremental trend increasing the reaction time, showing an oxidation of about 10% of the C6 of the chitin monomers at 24 h. The trend observed is in accordance with the one extrapolated from the FT-IR spectra.

The XRPD analyses are reported in Table 2 and were used to identify the influence of oxidation on chitin’s crystallographic organization. The diffraction patterns show that, along the [010] direction, a reduction in the cell parameter is observed, increasing the reaction time. This reduction is also associated with a decrease in crystallinity, revelated by a decrease in the crystal size. Conversely, along the [100] direction, the crystallinity and the dimension of the lattice parameter increased. This structural reorganization is likely due to the oxidation of C6 to a carboxylate group and a successive rearrangement of the hydrogen bond network in the crystal during the oxidation reaction.

An increase in the average distance among the (100) plane agrees with what was expected with the C6 oxidation. In β-chitin, the C6 hydroxyl group is pointed along the a-axis and is involved in hydrogen bonding with the carbonyl group of the amide of the adjacent polymeric chain. Increasing the degree of functionalization is expected to increase the average distance along this direction since the carboxylate will require more space compared to a primary alcohol. Despite that, the carboxylate may act as a hydrogen bond receiver from the N-H of the amide. The hydrogen bond associated with a carboxylate group is expected to be stronger and more stable compared to that of an alcohol. Moreover, carboxylate has lower mobility due to its carbon hybridization and its higher steric hinderance compared to a primary alcohol. The combination of stabilization, new potential interactions, and a decrease in the mobility of this group may lead to the increase in crystallinity along this crystallographic axis.

On the other hand, a shrinkage of the cell parameters was observed along the [010] direction. This direction is perpendicular to the polymeric chains and does not present any direct hydrogen bonding between adjacent chains. The oxidation of the C6 may lead to the formation of direct hydrogen bonding in between polymeric chains, such as new hydrogen bonds involving the C=O of the carboxylate group, leading to a shortening of their average interplanar distance. Simultaneously, the relatively low degree of functionalization achieved will create small variations or kinks of the chains along this distance where the carboxylate group is, leading to an increase in the overall disorder of the crystal.

Based on the XRPD results, none of the rearrangement observed induced a significant loss in crystallinity.

In conclusion, combining the degree of functionalization calculated from the ss-NMR spectra (Figure 2) and the relative intensity of the FT-IR bands (Figure S3c), the 24 h time point was selected as the optimal reaction time. In fact, at 24 h, the reaction still shows a relevant product yield (32 wt.%), while the product has the highest degree of functionalization, approximately 10% of the C6 of β-chitin, with no significant loss in crystallinity.

### 2.3. Optimization of the Oxidation Time for α-Chitin

In contrast to β-chitin, α-chitin shows a markedly different reactivity, coherent with what has previously been documented in the literature [5,15]. According to the turbidity measurements, the transmittance of the reaction batch decreases much more slowly compared to the β-chitin; see Figure S2. This suggests that α-chitin fibers are less accessible to ammonium persulfate, and defibrillation occurs over longer periods. At about 72 h, a complete dissolution of the chitin was observed. Basing on the turbidity measurement results, we decided to focus our attention on the following reaction times, 9 h, 24 h, 48 h, and 120 h, to sample along different positions of the turbidity profile. No time longer than 120 h were tested (even if it still showed a yield of about 40 wt.%.) since the reaction time would be too long and not compatible with practical applications.

A lower reactivity of α-chitin can also be deduced by FT-IR spectra analyses (Figure S3). As for β-chitin, an absorption band at 1730 cm^−1^ is observed in the oxidized samples. A ratio between the intensity of this signal and the one at 1073 cm^−1^ was calculated, as was carried out for the other polymorph. After 120 h, the relative intensity of the carboxylic acid band increases by only 27%, a value inferior to that obtained for β-chitin after 24 h of reaction (30%).

Compared to β-chitin, the FT-IR analyses on the oxidized α-chitin samples showed a smaller increment in oxidation in the first 24 h. No differences in the FT-IR relative intensity and yield (Figure 1a) were observed between 48 h and 120 h, suggesting the reaction reached a plateau, and the decrease in turbidity was probably due to slow solubilization processes.

The XRPD pattern was acquired for each of the α-chitin powder synthetized and the data are reported in Table 3 and Figure S5. Comparing the diffraction pattern, no significant variation was observed along the (110) or the (020) directions in the peak position. A mild increase in crystallinity was observed along the (020) plane, while no relevant trend in the crystallinity was identified along the (110) plane. The same result was also obtained by Jiang J. et al. [40], in which α-chitin is oxidized using the O_2_/Laccase/TEMPO system, testing various concentrations of TEMPO. From the XRD analysis, it is observed that the structure of chitin remains mostly unchanged. Here, too, a slight increase in crystallinity is observed, probably due to the removal of the water-soluble amorphous zones during oxidation. These results suggest that oxidation occurs on the chitin fiber surface [40].

We chose to test only the β-chitin to produce oxidized chitin hydrogels, as α-chitin requires a long oxidation time to achieve a good degree of functionalization.

### 2.4. Rheological Analysis

**Figure 3:**
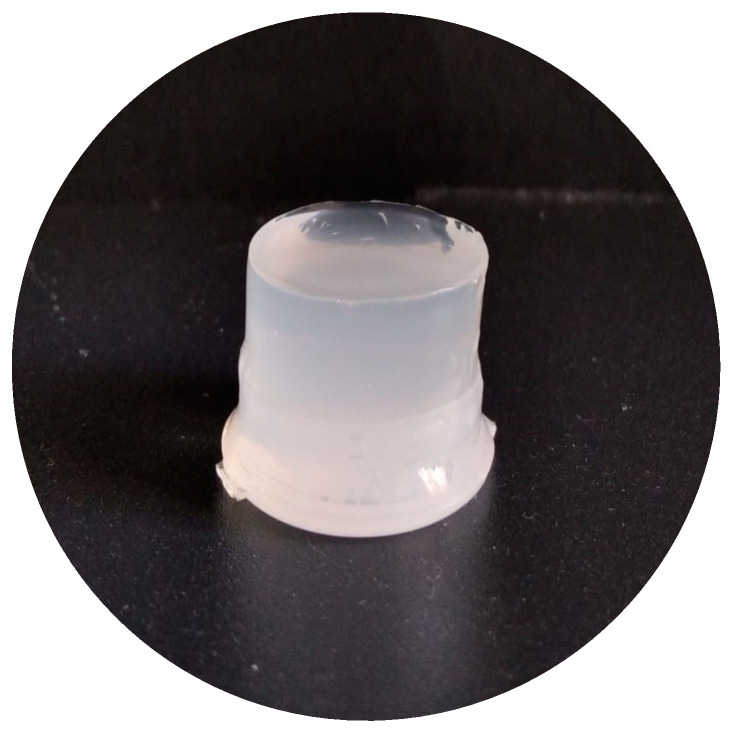
A β-chitin hydrogel camera picture. The hydrogel cylinder has a diameter of about 1 cm.

Following the procedure described in Section 3.9, a hydrogel was obtained from the β-chitin grains in the range of 600–150 µm, oxidized for 24 h. The hydrogel prepared (1 wt.% concentration) appeared as a homogeneous material with a soft consistency, as is visible in the picture in Figure 3.

With the aim to modulate its mechanical resistance and test its chemical stability, we exposed the hydrogel to diverse environments. The conditions studied were mostly the following two: in the presence of calcium ions, and under different pH values.

The samples were prepared as described in Section 3.9.1 and Section 3.9.2.

For the first study, various molar ratios between carboxylate groups and calcium ions (Ca^2+^) were tested. Different addition methods of Ca^2+^ were tested: (i) the surface addition of the calcium solution on the hydrogel, (ii) the mixing of the solution with the hydrogel, and (iii) the addition of Ca^2+^ before sonication. Among them, the surface addition method was selected, as it produced samples with a higher storage modulus (G′) and better reproducibility compared with (ii) (as reported in Table S1), while method (iii) showed the formation of aggregates and hydrogel clumps.

#### 2.4.1. Amplitude Sweep Tests

The viscoelastic behavior of the hydrogels in terms of storage (G′) and loss (G″) moduli, representing the elastic and the viscous portion of the viscoelastic behavior, respectively, was analyzed using rheological experiments. For all the tested samples, the G′ modulus is higher than the loss modulus G″, thus indicating that all the tested samples have a solid structure and can be classified as gels [41,42]. The linear viscoelastic region (LVE) indicates the range of strain where the sample is elastic and can recover its original state when the strain is removed. All the tested samples are very elastic, presenting a large LVE range and no crossover point (G′ = G″): none of the samples reaches the breaking point, where the material converts from solid to liquid.

Ca^2+^ were added in different molar ratios compared to the carboxylate groups, quantified by ss-NMR (See Section 2.2). The molar ratios studied were Ca^2+^: COO^−^ equal to 1:3; 1:1; 3:1, and 5:1. These concentrations were chosen to explore different binding ratios between the carboxylate and the Ca^2+^, assuming they would bind in a 1:1 or 1:2 ratio. Higher concentrations were studied to explore the presence of other binding sites.

**Figure 4:**
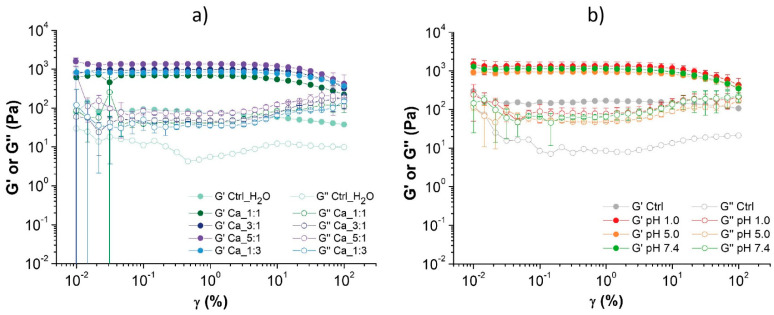
Amplitude sweep tests of the samples tested with (**a**) different molar ratios of Ca^2+^ and (**b**) different pH conditions. The standard deviation of the Ctrl and Ctrl_H_2_O sample was omitted for clarity and its full graph can be seen in Figure S6.

The amplitude sweep curves of all tested samples are shown in Figure 4, while the numerical values derived from the analyses of the Ca^2+^-treated samples are reported in Table 4. The data reveal that the incorporation of Ca^2+^ results in a notable enhancement of the stiffness of the gels, which may be the consequence of a probable physical crosslinking between the Ca^2+^ and the carboxylates present in the chain of the oxidized chitin [43]. Compared to the control, where the same volume of water was added, all the samples with Ca^2+^ have a storage modulus one order of magnitude higher and show a better reproducibility, with reduced standard deviation values compared to the control.

The increase in storage modulus rises with the increasing amount of added Ca^2+^, resulting in a mechanically stronger material [44].

A similar increase in storage modulus was observed by modifying the type of bivalent metal ion, using magnesium ions in a 3:1 ratio (800 Pa). Furthermore, slightly lower G′ values (690 Pa) was observed when a NaCl solution with the same ionic strength was added (Table S2).

Comparing with the results found in the literature, the hydrogels obtained here show a superior performance compared to other hydrogels from natural polymers. In the work by Syverud, Kristin, et al. (2015), a cellulose nanofibril hydrogel obtained with TEMPO achieves a storage modulus G′ of 275 ± 62 Pa when using a 0.98 wt.% dispersion [45]. Additionally, in the work by Cuomo et al. (2019), an increase in the strength of the alginate hydrogel is reported, correlated to the quantity of added Ca^2+^. However, using a 1 wt.% alginate solution, the maximum storage modulus reached is only 100 Pa [46]. Lower G’ values (about 10^2^ Pa) were also reported for gelatin gels (1 wt.%) blended with increasing amounts of chitosan (0.1–0.8 wt.%) with pH values between 3 and 4 [47]. Few examples of chitin gels were also reported, obtained by heating the polymer in ionic liquids. In these cases, the stiffness is highly dependent on the solvent used. Weak gels (G′ values in the range 10^1^–10^2^ Pa) [48] were obtained from IL (1-allyl-3-methylimidazolium bromide) for relatively high chitin concentrations (5–7 wt.%). G′ values slightly higher than ours (about 10^4^ Pa) were instead reported by Deng et al. (2020) for 1 wt.% chitin gels obtained in [BMIM]Ac (1-butyl-3-metlimidazolium acetate) [19].

Successively the stability of the hydrogel at different pHs, specifically 1.0, 5.0, 7.4, and 9.0, was tested. These pHs were chosen due to their biological relevance, as pH 1.0 is the pH associated with digestion (specifically in the stomach), 7.4 is generally considered a healthy physiological pH, and pH 5.0 is usually associated with inflammation. pH 9.0 was tested as an alkaline counterpart. In order to reproduce the hydrogel stability in a physiological environment, PBS was used as a buffer solvent at pH 7.4.

For this test, each hydrogel was immersed in a buffered solution for 24 h, while a hydrogel stored in milliQ water was measured as a control. At the end of this exposure, the hydrogels immersed in milliQ water and carbonate buffer at pH 9.0 were not tested because the gel lost its structure and dissolved completely. Due to the absence of a control experiment in the same condition, the initial hydrogel was used as the control experiment.

The remaining samples were measured for their rheological properties and the results of the amplitude test are shown in the Figure 4b, while the numerical data are reported in Table 5. The hydrogels immersed in pH 1.0, pH 5.0, and pH 7.4 show a storage modulus (G′) five times greater than the starting hydrogel used as a control, as seen in Figure 4b, but no significant difference in the rheological properties was observed among these conditions. A mild increase in the loss modulus was also observed in these samples compared to the control. Even in this case, no crossover points (G′ = G″) are detected: none of the tested samples reaches the breaking point in the tested shear strain range.

These results suggest that, within the oxidized chitin hydrogel, favorable interactions are formed at pH 1.0, 5.0, and 7.4. However, at very basic pH levels (pH 9.0), the interactions are so weak that they cause the collapse of the entire hydrogel structure. Indeed, at acidic pH levels, the deacetylated amines (pK_a_ 6.3) [5] of chitin protonate and can interact with the carboxylate ions (with a pK_a_ likely similar to that of alginate, 3.2 [49]) present in the polymer chains. Alternatively, since we do not know the actual pK_a_ of this compound, at basic pH levels, the carboxylic acids may deprotonate, and the electrostatic repulsion between polymeric chains, combined with the higher polymer solubility, may lead to the collapse of the hydrogel structure.

The collapse of the hydrogel in water, on the other hand, suggests that it is probably unstable at pHs much lower than pH 9. Acid pHs may stabilize the material, inducing positive interactions by protonation, while PBS may stabilize the structure by cation ionic bridges, as already observed by adding NaCl, MgCl_2_, or CaCl_2_. Considering the composition of PBS, the ion bridges formed are likely due to Na^+^ or K^+^. Specifically, in the previous section, it was observed that Na^+^ induced a toughening of the hydrogel, but its efficiency is lower compared to Ca^2+^. The higher G′ observed at pH 7.4 (PBS) is likely due to the abundance of Na^+^ in the PBS, which allowed us to saturate all the binding sites, maximizing the interactions in the hydrogel.

#### 2.4.2. Frequency Sweep Tests

Frequency sweep tests were carried out on the different hydrogel samples to describe their time-dependent behavior in the non-destructive deformation range (LVE). The shear strain value (ɣ = 0.1%) to perform the test was chosen within the LVE region, obtained from the amplitude sweep (see Section 3.10.1).

The results of the frequency sweep experiments (Figures S7 and S8) confirm the solid-like behavior of all the hydrogels (G′ > G″). Some of the samples show a crossover point at high frequencies (always ꙍ > 10 rad/s) where G′ < G″ and a probable breaking of the gel network occurs. The only samples where G′ and G″ are completely independent from the frequency and exhibit purely elastic properties [50] are the gel with Ca 5:1 and the one at pH 5.0, apart from the Ctrl, which has a lower value of G′. From this, it can also be deduced that the hydrogel right after the synthesis, Ctrl, is elastic, while the control sample with the addition of water, Ctrl_H_2_O, shows a viscoelastic behavior. This is coherent with the solubilization observed when fully immersed in water. In all samples, G′ exhibits a constant trend for the majority of the tested frequencies. This indicates the presence of a stable cross-linked network [51].

### 2.5. Structural Characterization

#### 2.5.1. Infrared Spectroscopy

The attenuated total reflectance (ATR) FT-IR spectra were performed on the freeze-dried hydrogels and used to investigate variations in the interactions in the hydrogel network in the different environments. In all samples, the same pattern of absorption bands was observed and only shifts and changes in relative intensity were observed.

**Figure 5:**
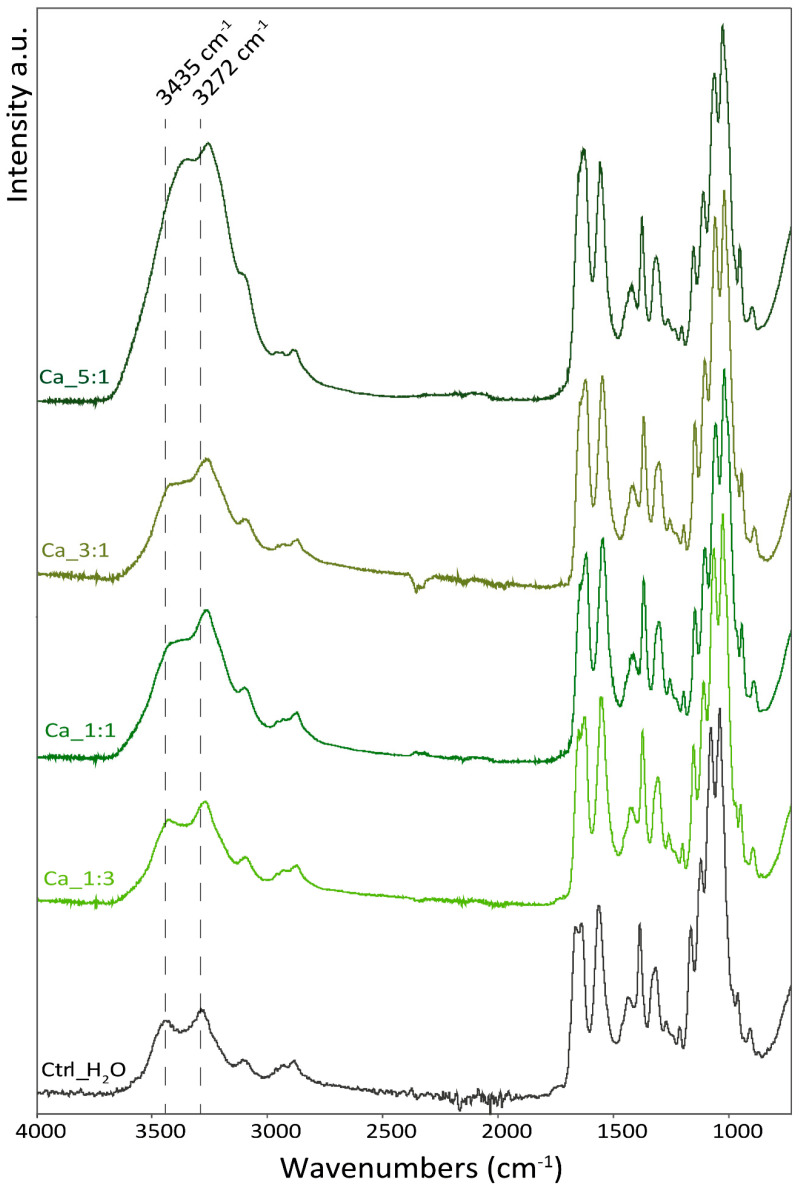
ATR-FTIR spectra of the samples with different Ca^2+^/carboxylate groups molar ratios. The dashed lines indicate the stretching motion of O-H (3435 cm^−1^) and N-H stretching (3281 cm^−1^) referred to the control sample (Ctrl_H_2_O).

In Figure 5, the spectra of the samples with an increased amount of Ca^2+^ are reported. The addition of Ca^2+^ was observed to induce changes in the O-H stretching (from 3435 to 3372 cm^−1^) and in the shape of the amide I band, which exhibits two peaks (at 1630 and 1655 cm^−1^), in the control that merge into a single peak as more Ca^2+^ were added. Additionally, a shift in the peak of C-O stretching (from 1029 to 1025 cm^−1^ and from 1067 to 1064 cm^−1^) and N-H stretching is observed (from 3099 to 3108 cm^−1^; from 3280 to 3255 cm^−1^). No shift was observed in the absorption bands associated with the carbon backbone of the polymer (i.e., sugar ring vibration).

The shifts observed are mostly associated with functional groups involved in hydrogen bonding as a donor or receiver. According to that, Ca^2+^ ions seem to interact in between polymeric chains and mediate hydrogen bonding between the chitin polymeric chains involving hydroxyl groups, and the amide N-H and carbonyl group. This intercalation does not appear to interfere with the polymer chain structure which appears unaltered, meaning no intramolecular difference in the degree of freedom of the polymeric chains is observed.

**Figure 6:**
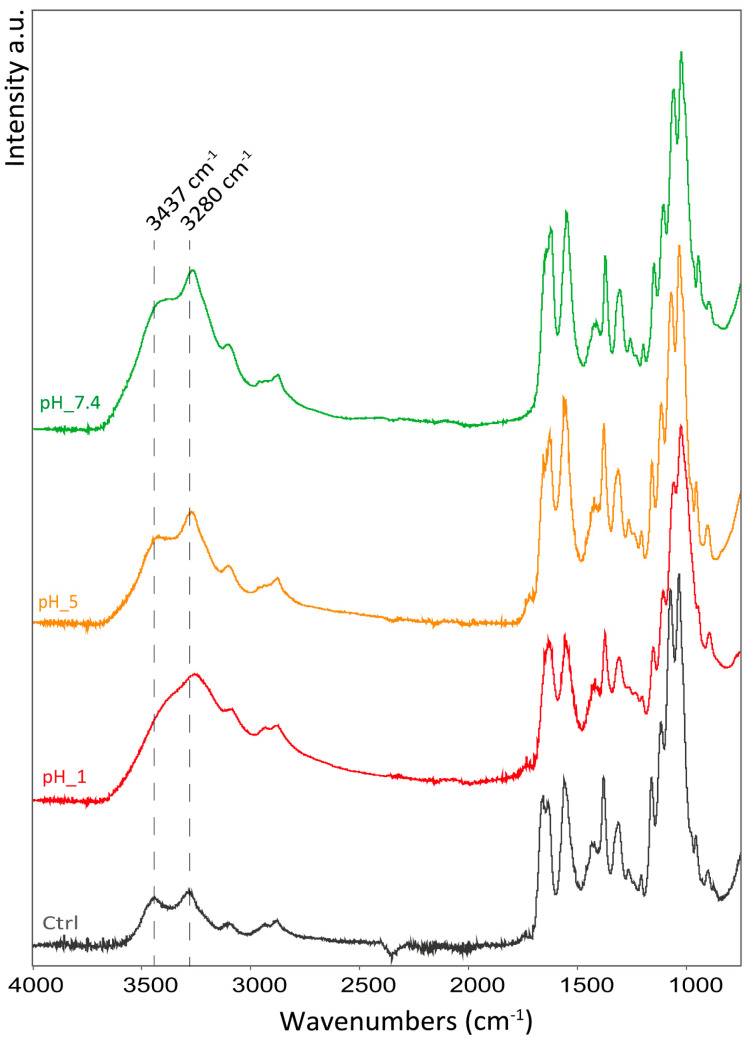
ATR-FTIR spectra of samples in different pH conditions. The dashed lines indicate the stretching motion of O-H (3437 cm^−1^) and N-H stretching (3280 cm^−1^) referred to the control sample (Ctrl).

In the hydrogels stored at different pH conditions, the O-H stretching band shifted to lower wavenumbers (from 3280 to 3255 cm^−1^) along with the N-H stretching (from 3094 to 3090 cm^−1^) as the pH decreases. Along with this shift, the two bands tend to merge into one; see Figure 6.

The amide I band exhibits the same trend observed in the hydrogels treated with calcium where the control shows a band split into two peaks that merge into one as the pH decreases.

The pH variation also affects the ring vibration, as evidenced by the shift of the ring stretching band from 894 to 900 cm^−1^, increasing the pH. Other bands associated with the polymer backbone do not appear to show significant shifts (i.e., asymmetric bridge oxygen stretching, or asymmetric in phase ring stretching).

Contrary to the Ca^2+^-treated hydrogels, a shift in the CH_2_ bending and CH_3_ deformation was observed from 1429 cm^−1^ in the control to about 1419 cm^−1^ for all the pH-treated samples. No other significant shift was observed in other apolar moieties.

As observed with the hydrogel treated with Ca^2+^, an increase in the hydrogel rheological properties is associated with shifts to lower wavenumbers in the functional groups involved in hydrogen bonding (O-H, N-H, and C=O). This suggests a shortening of the hydrogen-bonding interactions, leading to more polarized functional groups and higher interactions between polymeric chains. Along with that, pH appears to also force additional interactions between apolar groups that affects the mobility of the chitin ring. This interaction is likely positively driven by the increment in the polarity and ionic strength of the solution.

All peak assignments are provided in the supporting information Table S3 [52].

#### 2.5.2. X-ray Powder Diffraction Analysis

X-ray powder diffraction (XRPD) analysis was performed on the freeze-dried hydrogels to investigate their structural organization.

**Figure 7:**
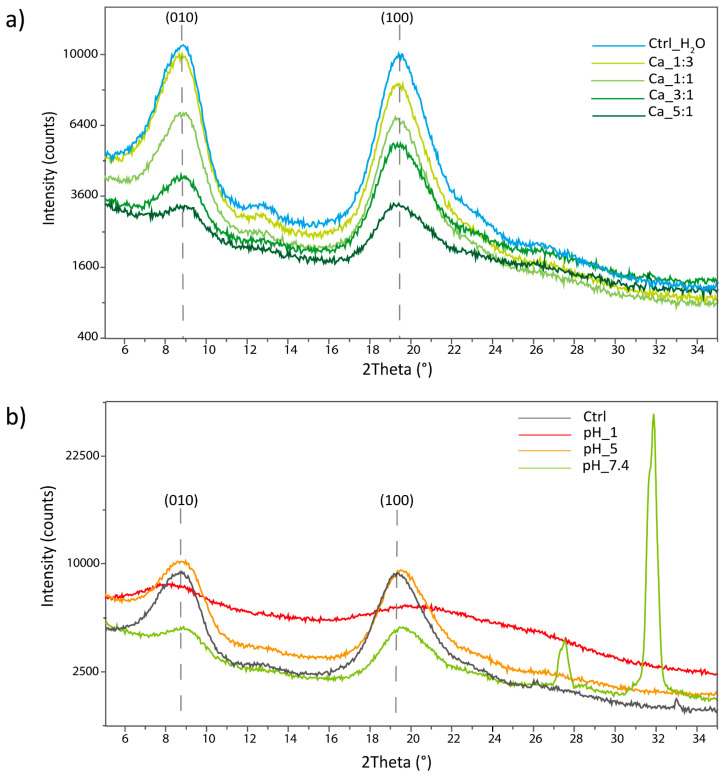
XRPD analyses on freeze-dried hydrogels treated (**a**) with different carboxylate group/calcium ion molar ratios (**b**) in different pH conditions. Chitin’s diffraction peaks are a convolution of different signals; the Miller indices reported refer only to the most intense signals which account for most of the signal intensity. In the sample treated at pH 7.4, two additional peaks are visible; those peaks have been attributed to inorganic salts derived from traces of PBS in the hydrogel during freeze-drying.

In the XRPD analyses of the hydrogels obtained with different Ca^2+^ additions, reported in Figure 7a, an overall decrease in the crystallinity was observed, increasing the Ca amount. This was observed as a decrease in the overall signal intensity; the XRPD were acquired on a similar amount of the sample.

A reduction in the unit cell parameter along the [010] direction was observed, increasing the Ca^2+^. Indeed, the peak corresponding to the (010) reflection shifts from a value of 8.82° in the control sample treated with water to a value of 9.06° in the sample treated with Ca 5:1. This corresponds to a decrease in interplanar distance of 0.26 Å. The shift was associated with an increase in the crystallinity of this diffraction peak, visible as an increase in the crystal size. The results are reported in Table 6.

An increment in the a-axis unit cell parameter (0.02 Å), from 19.48° to 19.39°, was observed along the [100] direction, increasing the Ca^2+^. In this case, a reduction in the crystallinity was observed along this direction. In this direction, an initial increase in crystallinity was observed, moving from the control to the sample treated with Ca 1:3 or 1:1.

The results suggest that Ca^2+^ ions intercalate in the crystal structure, disrupting its order and decreasing the overall polymer crystallinity. This intercalation is likely initially due to the chelation of the Ca^2+^ by the carboxylate groups, and then probably affects different non-specific binding sites. A similar effect has also been reported in the preparation of a hydrogel starting from carboxymethylcellulose (CMC) crosslinked with calcium ions. In their work, the XRD analysis showed a crystallinity index (CI) of 32.77% for the CMC which becomes equal to only 4.24% when calcium is added [53].

In our case, this interaction is probably occurring along the a-axis (between the two planes of the sugar rings of adjacent polymeric chains) where we theorized the carboxylic group to be mostly located and involved as a hydrogen-bonding acceptor with the N-H of the amide in the adjacent chain. Ca^2+^ is probably inserting in between the two parts of this hydrogen bond, inducing an enlargement of the cell parameter and introducing big defects in the crystal structure. The insertion of calcium ions along the a-axis in between polymeric chains could induce important modifications in the chain-to-chain interaction along the b-axis (where polymeric chains are positioned side-to-side). The presence of Ca^2+^ could seize the carboxylate, eliminating the kinks and defects accumulated along this axis after the functionalization. On the other hand, the presence of a positive charge is probably acting as a bridge between the chains, inducing a contraction of the unit cell along this axis. Due to a lower steric hindrance along this plane and a higher interaction, the cell shrinks and becomes more ordered along this direction.

The XRPD acquired on the hydrogels exposed to different pHs are reported in Figure 7b. This set of samples showed a less clear trend compared to the Ca^2+^-treated ones.

The overall intensity appeared to be comparable between the control and the sample at pH 5, while the sample at pH 7.4 showed an intensity intermediate to that of the samples treated with Ca 3:1 and 5:1. Finally, the sample treated at pH 1 showed the lowest overall crystallinity among all the hydrogel tested.

The same trend is also observed along the [100] direction where a reduction in the unit cell parameter is observed, moving from the control to pH 5, pH 7.4, and pH 1. The shift was also associated with a reduction in the crystal size. The results are reported in Table 7.

Along the [010] direction, a contraction of the cell parameter is observed at pH 5 and 7.4 along with an increase in crystal size. At pH 1, instead, a strong expansion of the unit cell and a decrease in crystallinity is observed along this direction.

In the sample at pH 7.4, two additional signals were observed at 27.51° and 31.87°. These signals were attributed to inorganic salts derived from the PBS solution entrapped in the hydrogel.

The XRPD results suggest a different interaction is governing the aggregation at pH 7.4 and 5 since a contraction is observed along the [100] direction, suggesting a positive contribution by the presence of ions to the interchain interactions. The carboxylate protonation in acid environment could, in fact, induce a change in the hydrogen-bonding pattern along the a-axis. It would likely interact with the C=O of the amide of the adjacent polymeric chain, restoring the native hydrogen bond pattern of β-chitin. Most likely, both ions’ interactions, coherent with the observations in the (010) plane, and protonation are occurring, leading to the increase in disorder observed.

Contrary to all the previous samples, the sample at pH 1 appears strongly altered, up to almost complete amorphization. Due to that, the results concerning the peak position or the crystal size cannot be considered reliable. This strong alteration may be the result of a complete protonation of the carboxylic acids and/or the co-ordination of positive charges in the crystal in non-specific binding sites. In fact, β-chitin has already been reported as strongly swellable in acid pHs [7,54] and this amorphization may have been guided to the same interactions.

#### 2.5.3. Scanning Electron Microscopy Analyses

Scanning electron microscopy (SEM) was used to analyze the morphology and aggregation of the freeze-dried hydrogels. After freeze-drying, all the samples retained their shape as hydrogels and showed a soft consistency; see Figure S9. The hydrogel treated at pH 1, instead, showed a strong shrinkage and a brittle consistency.

**Figure 8:**
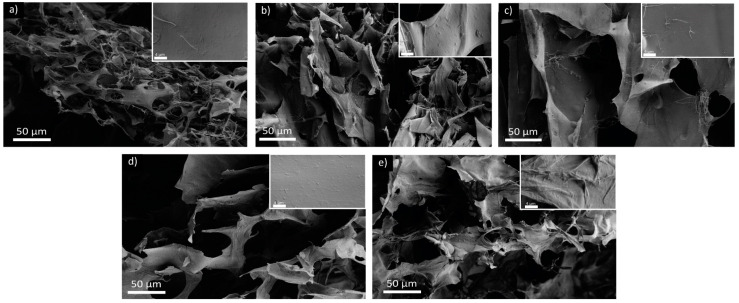
SEM images of the freeze-dried hydrogels: (**a**) Ctrl_H_2_O; (**b**) Ca_1:3; (**c**) Ca_1:1; (**d**) Ca 3:1; and (**e**) Ca_5:1. For each condition, an insight with a higher magnification is reported.

Observing the samples with SEM, all samples showed a porous structure with smooth sheet-like walls and filaments of fibers embedded in the matrix. The increment in the Ca^2+^ addition was observed to be associated with a thickening of the pore walls and a reduction in the free fiber presence (Figure 8).

**Figure 9:**
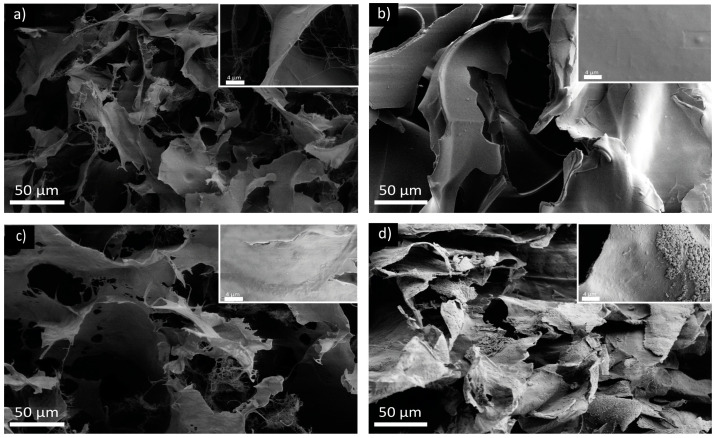
SEM images of the freeze-dried hydrogels exposed to different pHs: (**a**) Ctrl; (**b**) pH_1; (**c**) pH_5; and (**d**) pH_7.4. For each condition, an insight with a higher magnification is reported.

A similar wall thickening and fiber reduction was observed also in the samples exposed to different pHs, as is visible in Figure 9. This effect followed the same trend observed in the XRPD, control sample, pH 5, pH 7.4, and, finally, pH 1. The latter showed the thickest walls among all samples and exhibited sharper and more delineated fractures.

The sheet-like morphology observed could be an artifact of the sample freezing and may not be representative of the hydrogel organization in solution. Despite that, the SEM analyses suggests that an increase in the interactions of the hydrogel occurs, increasing the cation concentration in solution—we see a thickening of the laminar structures. This thickening is probably due to the aggregation of loosely bound fibers, which are then forced to interact with the hydrogel network.

### 2.6. Discussion on Hydrogel Tunability

The characterizations performed on the hydrogels exposed to different environments showed how the rheological properties and the structure of the material can be rearranged based on diverse triggers. Such a rearrangement can increase the storage modulus of the gel from 5 to 10 times. These triggers are generally related to positively charged chemical species, such as metal cations or hydrogen ions.

These cations interact with the polymeric chains and even intercalate in the chitin’s crystal structure. The cations seem to interact with groups generally involved in hydrogen bonding, namely, alcohols, amines, and amides (both N-H and C=O). A strong interaction is also likely occurring with the carboxylic group. These interactions probably see the cation involved in the dipolar interaction between these groups.

When the ions intercalate in the crystalline regions, this seems to happen along the a-axis, in between the sugar ring plane of the polymeric chains, causing a decrease in crystallinity. When Ca^2+^ ions are intercalating, an expansion of the crystallographic unit cell in this direction is observed. On the other hand, a contraction is observed when monovalent cations, like Na^+^, K^+^, or H^+^, interact with the hydrogel crystal units. This contraction is probably the reason why the CH_2_ bending and the sugar ring stretching are affected.

Simultaneously, a contraction over the b-axis, where the polymeric chains are arranged side-to-side, is observed in all the conditions tested, along with an increase in crystallinity. This suggests an increment of the interactions over this direction, generally presenting very little direct interactions in β-chitin. Overall, the intercalation of cations in the crystal is associated with a decrease in crystallinity due to a disruption of the crystal structure order; this loss in crystallinity increases, increasing the ion concentration in solution.

The morphological examination of the freeze-dried hydrogels suggests an increase in the hydrogel network interaction, increasing the cation presence. It also shows how the fibrils in the hydrogel are only partially interacting in the control, and then increase their interaction when exposed to cations. This observation suggests that the cations induce the formation of additional linkage in the matrix, or favor the formation of the one already possible, forcing new fibrils to aggregate in the hydrogel network.

It is worth mentioning that the frequency sweep tests identified four samples with viscoelastic behavior instead of an elastic one. Except for one of the controls, the samples exhibiting a viscoelastic behavior are the ones exposed to the lowest and highest ion amounts—respectively, the Ca 1:3, the sample in PBS (pH 7.4), and the one at pH 1. This suggests an optimal ionic exposure that maximize the properties of the hydrogel, giving it an elastic behavior. This condition varies when different ions are used. Such a change in the rheological properties is probably due to an excessive aggregation of the fibrils, as visible in the SEM of the sample at pH 1, which confer a more brittle behavior.

## 3. Materials and Methods

### 3.1. Materials

The β-chitin flakes from the squid pens of *Loligo vulgaris* were purchased from BioLog Heppe^®^ GmbH (Landsberg, Germany). The α-chitin powder from shrimp shells was purchased from Glentham Life Sciences (Planegg, Germany). All other chemical reagents were purchased from Merck and used without any additional purification.

### 3.2. Chitin Ball Milling

The initial β-chitin powder was ground with a ball mill 8000 Mixer Mill^TM^ SPEX CertiPrep^TM^ (Metuchen, NJ, USA). The jar used has a diameter of 3.85 cm and height 5.70 cm. The grinding was carried out with three small spheres with a diameter of 0.62 cm and three spheres with a diameter of 1.27 cm. Both the jar and the spheres are made of hardened steel. Then, 2 g of powder were ground at a time for 10 min. Subsequently, both β-chitin and α-chitin were sieved through 600, 150, and 45 μm sieves (GIULIANI Tecnologie srl, Turin, Italy) to separate different powder meshes.

### 3.3. X-ray Powder Diffraction Analysis

X-ray diffraction patterns were collected using a PanAnalytical X’Pert Pro diffractometer equipped with a multiarray X’Celerator detector using Cu Kα radiation generated at 40 kV and 40 mA (λ = 1.54056 Å).

The diffraction patterns were collected in the 2θ range between 5° and 35° with a step size (Δ2θ) of 0.05° and time per step of 123 s.

The peak maximum and full width at half maximum (FWHM) measurements were calculated using the PanAnalytical X’Pert Data Viewer software (version 1.2d). The value was then used to determine the crystallite size (d) using the Sherrer equation.

The crystal index (CI) was determined as a percentage using the following equation [55,56]:

CI = [(I_cryst_ − I_am_)/I_cryst_] × 100

where I_cryst_ is the intensity in counts at the maximum of the reflex and I_am_ is the intensity of amorphous diffraction at 2θ ≅ 14°.

### 3.4. Chitin Oxidation

For the oxidation reaction, 0.5 g of chitin powder (600–150 µm) were added in 50 mL of a 45 wt.% ammonium persulfate solution (APS) at 40 °C under stirring for diverse reaction times in a round bottom flask. For gel production, β-chitin was stirred for 24 h.

After that, the solution appears as an opalescent dispersion. The material in suspension was collected and washed with deionized water by consecutive centrifugation with an Eppendorf Centrifuge 5810 R at 8000 rpm for 5 min until it reached neutrality.

The powder was then resuspended in 30 mL of milliQ water and left under magnetic stirring overnight, to eliminate possible reaction residues. The next day, the powder was centrifuged, as was carried out previously, and resuspended in about 15 mL of milliQ water, frozen in the freezer, and desiccated by freeze-drying using a FreeZone 1 (Labconco Corp., Kansas City, MO, USA). The product was conserved dry at room temperature.

### 3.5. ss-NMR Measurements

The ^13^C solid-state nuclear magnetic resonance (ss-NMR) experiments were performed on a Bruker 700 MHz Wide Bore spectrometer equipped with a 4 mm CP MAS X/Y/H DVT probe in a double-resonance H-X configuration. The analyses were performed operating at 260 K and MAS speed of 12 kHz. The CP contact time was 2 ms, and the recycle delay was 10 s. A typical number of 512 or 2048 scans were acquired for each spectrum.

The degree of functionalization (%*DF*) was calculated, setting the total integral for C3, C4, and C5 to 3 and measuring the C6 integral (*I_t_*). The %*DF* was then calculated using the following formula:

$$\%DF=(1-\frac{I_{t}}{I_{t0}})\times 100$$

where I*_t_* is the integral of the C6 signal attributed to the various oxidized chitin samples and it is the one of the initial chitins. The NMR signal attribution is reported in Figure S4 [15,57].

### 3.6. UV–Visible Spectroscopy

Spectrophotometric measurements were carried out with a UV–vis spectrophotometer (Varian Cary 300 Bio UV–Visible Spectrophotometer, Santa Clara, CA, USA) using a spectral range of 300–900 nm, scan rate of 600 nm·min^−1^, with a resolution of 1 nm. Turbidity measurements were measured at 500 nm.

### 3.7. FTIR-Spectroscopy

The infrared spectra were collected using a Fourier-transform infrared spectroscopy (FTI) (Thermo Scientific Nicolet iS10, Waltham, MA, USA) and processed with Omnic software 9.8.286 (Thermo Electron Corp., Woburn, MA, USA).

The samples were prepared in KBr pellets (1 wt.% of sample) and the spectra were collected with 2 cm^−1^ resolution and 70 scans.

The intensity of each peak was obtained by correcting the intensity on the baseline [30]. The oxidation degree was then evaluated by calculating the ratio between the carboxylic acid peak (1730 cm^−1^) and the most intense peak of the glucosidic ring which falls at 1074 cm^−1^ [58].

### 3.8. Product Yeld

The product yield was calculated based on the initial mass (*mg_i_*) of chitin powder and the final mass (*mg_f_*) obtained from the oxidation reaction and calculated using the following formula:

$$product\;yield=\frac{{mg}_{f}}{{mg}_{i}}\times 100$$

### 3.9. Hydrogel Preparation

Oxidized β-chitin powder (βchitOx) was dispersed in milliQ water to obtain a 1 wt.% dispersion. This was vigorously stirred using a magnetic stirrer for about 2 h at room temperature.

Once no visible aggregates were observed by the naked eye, the pH of the dispersion was between 3 and 4. A required volume of a 1 M NaOH solution was added to the dispersion to bring the pH between 8 and 9.

The obtained dispersion was divided into 2 mL aliquots inside sterilin tubes (Thermo Scientific™ Sterilin™ 7 mL Polystyrene Bijou Containers, Waltham, MA, USA) and sonicated at 10% amplitude with a tip sonicator (Branson Ultrasonics^TM^ Brookfield, WI, USA; Sonifier 250; 20 kHz) equipped with a semimicro tip, with the container immersed in an ice bath to prevent overheating. The sonicated β-chitOx dispersion was then left to rest to obtain the βchitOx-hydrogel. Since only one type of unaltered (with Ca^2+^ or pH) hydrogel was prepared in this study, we will refer to this sample as hydrogel or control.

#### 3.9.1. Metal Ion Addition to the Hydrogel

To investigate the effect of Ca^2+^ on the hydrogel, different CaCl_2_ solutions were prepared at concentrations of 16.6, 50, 150, and 250 mM. A volume equal to 196 μL of these solutions was used to obtain molar ratios of 1:3, 1:1, 3:1, and 5:1 with the carboxylate groups formed after the reaction of oxidation (quantified by ss-NMR measurement). The tests on Mg^2+^ and Na^+^ were performed using MgCl_2_ and NaCl solutions.

Different addition methods were tested:

- Surface addition: Allowing the solution to drip from the walls of the sterilin tubes containing the hydrogel.
- Mixed addition: Inserting the solution into the hydrogel, and then mixing with the pipette tip. This process disrupts the hydrogel to better blend the solution.
- Calcium solution addition before the sonication step.

The hydrogel is then left to rest for 24 h to allow the Ca^2+^ to diffuse homogenously.

#### 3.9.2. Study of pH Influence on the Hydrogel

The sterilins containing the hydrogels were immersed in beakers containing 90 mL of solutions at different pH values: 1 (0.1 M HCl); 5 (10 mM citrate buffer Na_3_C_6_H_5_O_7_/C_6_H_8_O_7_); 7.4 (phosphate buffered saline, PBS); 9 (10 mM carbonate buffer NaHCO_3_; Na_2_CO_3_); and MilliQ water used as control. The sterilins were left in solution with a mild stirring on a rocking table for 24 h; along this time, the buffer solution was changed twice to maintain a constant environment.

### 3.10. Hydrogel Characterization

#### 3.10.1. Rheological Properties

The rheological properties of the different hydrogels were assessed using an Anton Paar (Graz, Austria) MCR102 rheometer. The tested hydrogels (2 mL) were directly prepared in 7 mL Sterilin™ polystyrene containers (Thermo Scientific™, Waltham, MA, USA) which fit in the rheometer. A cup and vane geometry was used, setting a gap of 2.1 cm. Oscillatory amplitude sweep experiments (ɣ: 0.01–100%) were performed using a constant angular frequency of 10 rad·s^−1^. The frequency sweep experiments were performed at a constant shear strain of ɣ = 0.1% (within the LVE region) with an increasing angular frequency (ꙍ) from 0.1 to 100 rad·s^−1^. All tests were performed at a fixed temperature of 23 °C, controlled by an integrated Peltier system.

#### 3.10.2. FTIR-Spectroscopy

The hydrogels were analyzed using the FTIR equipment described in Section 3.7. The hydrogels were frozen with liquid nitrogen, and then dried by freeze-drying using a FreeZone 1 (Labconco Corp., Kansas City, MO, USA). The dry samples were measured in attenuated total reflectance (ATR) mode using 1 cm^−1^ resolution and 200 scans. The data analysis was then performed using the OMNIC software 9.8.286.

#### 3.10.3. X-ray Powder Diffraction Analysis

For the X-ray powder analysis of the freeze-dried hydrogels (see Section 3.10.2), the same instrumentation described in Section 3.3 was used. The diffraction patterns were collected in the 2θ range between 5° and 35° with a step size (Δ2θ) of 0.05° and a time per step of 360 s.

#### 3.10.4. Scanning Electron Microscopy Images

For the scanning electron microscopy (SEM) imaging, the samples were dried, as reported; see Section 3.10.2 (Figure S8) A piece of the dry samples was then cut with a scalpel, glued on a carbon tape, and coated with 10–20 nm of gold prior analyses. The images were collected with a SEM Zeiss LEOc1530 FEG using a voltage of 5 kV and an aperture of 20 μm.

## 4. Conclusions

In this study, we prepared tunable chitin hydrogels, exploring in detail, optimizing, and customizing a known synthetic process. The grain size of the starting material (between 600 μm and 150 μm) and the reaction time (24 h) were optimized to achieve highly crystalline oxidized β-chitin fibrils (having a %DF of 10%) with a yield of a 32 wt.%. Different reaction conditions were also tested on α-chitin, which exhibited a lower reactivity and a much longer reaction kinetic. Structural characterization (FT-IR and XRPD) was performed on all the reaction products to identify how the functionalization affects the structure of the biopolymer.

Successively, a hydrogel was obtained from the oxidized β-chitin. Such a hydrogel exhibited tunable rheological properties (up to a 10-times increment in G′) when exposed to cations (Ca^2+^, Mg^2+^, Na^+^, or H^+^), which induce structural rearrangements. The initial gelation is induced by a few minutes of sonication and occurs along the time, but it can be fastened if cations are present. Such a process could be compatible with an application as injectable gels for wound sealing or surgery since the physiological ion concentration would be enough to induce the formation of a tough hydrogel (as observed in PBS). Moreover, the hydrogel is stable in physiological environments, both healthy (pH 7.4), inflamed or unhealthy [59,60] (pH 5), and those connected to digestion (pH 1). This allows its use as a biomedical material for application with almost every human tissue. This also makes them a material of potential interest for the development of wound dressings. On the other hand, the instability of these hydrogels in alkaline environments and pure water make their disposal extremely easy and eco-friendly. Potentially, the hydrogel could even be dissolved at pH 9, and the fibrils could be recovered and recycled to produce a new hydrogel. Such a solubility could also be exploited to deliver biologically active compounds into the stomach of animals having an alkaline stomach pH, such as pests, like insects, or economically relevant animals, like sea urchins [61,62].

In conclusion, these negatively charged β-chitin fibrils could act as complementary materials to positively charged chitosan fibers. Additionally, their potential to obtain acid-stable tough hydrogels, that, alternatively, could be reinforced using metal cations, could represent an alternative to the structurally similar alginate hydrogels. This study sheds light on an alternative material with peculiar properties that could represent an important resource for the development of future technological materials, especially in the biomedical field.

## Figures and Tables

**Table 1:** Diffraction angles and crystallite sizes (d) of the main reflections for the various grain sizes of α-chitin and β-chitin. The standard deviation of the measurements is reported in parentheses. Chitin’s diffraction peaks are a convolution of different signals, the Miller indices reported refer only to the most intense signals which account for most of the signal intensity.

		600–150 μm			150–45 μm			<45 μm		
Sample		2Ɵ (°)	d (nm)	CI (%)	2Ɵ (°)	d (nm)	CI (%)	2Ɵ (°)	d (nm)	CI (%)
α-chitin	(020)	9.26 (0.01)	7.05 (0.07)	79.7 (0.4)	9.16 (0.06)	7.2 (0.2)	78.0 (0.2)	9.09 (0.03)	7.0 (0.2)	70.7 (0.7)
	(110)	19.22 (0.01)	4.82 (0.03)	83.9 (0.2)	19.13 (0.06)	4.8 (0.2)	81 (1)	19.07 (0.04)	4.48 (0.03)	75.1 (0.7)
β-chitin	(010)	8.2 (0.1)	4.04 (0.09)	69 (1)	8.38 (0.06)	3.78 (0.08)	59 (3)	8.07 (0.01)	3.52 (0.07)	46 (1)
	(100)	19.7 (0.1)	2.34 (0.08)	68.7 (0.4)	19.92 (0.03)	2.17 (0.01)	61 (1)	19.62 (0.01)	1.78 (0.01)	49.5 (0.5)

**Table 2:** Diffraction angles and crystallite sizes (d) of the main reflections for the various oxidation time of β-chitin. The standard deviation of the measurements is reported in parentheses. Chitin’s diffraction peaks are a convolution of different signals; the Miller indices reported refer only to the most intense signals which account for most of the signal intensity.

	(010)			(100)		
Sample	2Ɵ (°)	d (nm)	CI (%)	2Ɵ (°)	d (nm)	CI (%)
Control	8.21 (0.04)	4.0 (0.1)	69 (1)	19.70 (0.01)	2.4 (0.1)	68.7 (0.4)
3 h	8.32 (0.02)	3.74 (0.08)	74 (2)	19.50 (0.05)	2.60 (0.09)	73 (3)
6 h	8.24 (0.01)	3.65 (0.08)	76 (2)	19.44 (0.01)	2.70 (0.04)	73 (3)
9 h	8.62 (0.09)	3.5 (0.3)	65 (5)	19.35 (0.07)	2.73 (0.02)	71 (1)
24 h	8.63 (0.07)	3.3 (0.1)	66 (3)	19.26 (0.04)	2.78 (0.07)	75.4 (0.4)

**Table 3:** Diffraction angles and crystallite sizes (d) of the main reflections for the various oxidation time of α-chitin. The standard deviation of the measurements is reported in parentheses. Chitin’s diffraction peaks are a convolution of different signals; the Miller indices reported refer only to the most intense signals which account for most of the signal intensity.

	(020)			(110)		
Sample	2Ɵ (°)	d (nm)	CI (%)	2Ɵ (°)	d (nm)	CI (%)
Control	9.26 (0.04)	7.2 (0.1)	79.7 (0.4)	19.25 (0.01)	5.23 (0.04)	83.9 (0.2)
9 h	9.18 (0.07)	7.6 (0.1)	83.6 (0.4)	19.13 (0.07)	5.1 (0.1)	89.2 (0.8)
24 h	9.21 (0.04)	8.1 (0.4)	83 (2)	19.13 (0.01)	5.3 (0.1)	89.8 (0.8)
48 h	9.21 (0.04)	8.1 (0.4)	83.0 (0.1)	19.2 (0.1)	5.37 (0.06)	91.6 (0.9)
5 gg	9.25 (0.09)	8.0 (0.6)	79.9 (0.2)	19.22 (0.05)	5.0 (0.3)	91.0 (0.2)

**Table 4:** Table shows the values of G′ and G″ calculated at a shear strain value equal to 0.046%.

Sample	Storage Modulus G′; (ɣ = 0.046%)	Loss Modulus G″; (ɣ = 0.046%)
	[Pa]	[Pa]
Ctrl_H_2_O	80 ± 50	20 ± 10
Ca_1:3	820 ± 30	30 ± 10
Ca_1:1	690 ± 30	40 ± 20
Ca_3:1	940 ± 50	50 ± 20
Ca_5:1	1100 ± 300	60 ± 40

**Table 5:** Table shows the values of G′ and G″ calculated at a shear strain value equal to 0.046%.

Sample	Storage Modulus G′; (ɣ = 0.046%)	Loss Modulus G″; (ɣ = 0.046%)
	[Pa]	[Pa]
Ctrl	200 ± 100	20 ± 20
pH 1	1300 ± 400	50 ± 10
pH 5	950 ± 60	70 ± 10
pH 7.4	1150 ± 30	50 ± 30

**Table 6:** Diffraction angles and crystallite sizes (d) of the main reflections in the diffractograms obtained for the hydrogels containing different Ca^2+^/carboxylate group molar ratios. Chitin’s diffraction peaks are a convolution of different signals; the Miller indices reported refer only to the most intense signals which account for most of the signal intensity. * The values reported for the Ca_5:1 should be considered indicative, given the low quality of the diffraction peaks.

	(010)			(100)		
Sample	2Ɵ (°)	d (nm)	CI (%)	2Ɵ (°)	d (nm)	CI (%)
Ctrl_H_2_O	8.82	3.59	73	19.48	3.08	72
Ca_1:3	8.78	3.59	74	19.41	3.21	70
Ca_1:1	8.87	3.69	69	19.41	3.17	68
Ca_3:1	8.88	3.94	51	19.43	2.89	62
Ca_5:1 *	9.06	4.38	42	19.39	2.77	43

**Table 7:** Diffraction angles and crystallite sizes (d) of the main reflections in the diffractograms obtained for the hydrogels treated in different pH conditions. Chitin’s diffraction peaks are a convolution of different signals; the Miller indices reported refer only to the most intense signals which account for most of the signal intensity. * The values reported for the pH_1 sample should be considered indicative, given the low quality of the diffraction peaks.

	(010)			(100)		
Sample	2Ɵ (°)	d (nm)	CI (%)	2Ɵ (°)	d (nm)	CI (%)
Ctrl	8.71	3.59	73	19.39	3.14	73
pH 1 *	8.30	2.48	52	20.67	0.97	52
pH 5	8.81	3.65	67	19.58	3.07	64
pH 7.4	8.94	4.15	31	19.64	2.91	13

## Data Availability

The data can be made available upon request to the corresponding author.

## Supplementary Materials

The following supporting information can be downloaded at: https://www.mdpi.com/article/10.3390/md22040164/s1, the full characterization of the oxidized chitins, missing rheological data or characterization of the hydrogel not reported in the main text (including frequency sweep tests), and the interpretation of the FT-IR bands.
