# Supplementary material for: Tunable Oxidized-Chitin Hydrogels with Customizable Mechanical Properties by Metal or Hydrogen Ion Exposure
Source: Mar Drugs. 2024 Apr 3;22(4):164. doi: 10.3390/md22040164 (PMC11051383; doi:10.3390/md22040164)
Supplement: Supplementary file 1 [file marinedrugs-22-00164-s001.zip › marinedrugs-2919293-supplementary.pdf]

Supplementary Information

Tunable oxidized-chitin hydrogels with customizable mechanical properties by metal or hydrogen ion exposure

A. Mucaria, D. Giuri, C. Tomasini, G. Falini and D. Montroni*

Department of Chemistry "Giacomo Ciamician", University of Bologna, via F. Selmi 2, 40126 Bologna, Italy

Index

Figure S1. XRPD diffractograms - powder grain size optimization Page 2

Figure S2. Uv-visible measurements Page 3

Figure S3. α and β-chitin FT-IR spectra Page 3

Figure S4. ss-NMR Page 4

Figure S5. XRPD diffractograms-time optimization spectra Page 5

Figure S6. Amplitude sweep test of the ctrl and ctrl_H_2Osample Page 5

Figure S7. Frequency sweep Calcium ions hydrogels Page 6

Figure S8. Frequency sweep hydrogels in different pH conditions Page 7

Figure S9. Image of hydrogels after freeze-drying Page 7

Table S1. G' and G'' values of the various addition methods tested Page 8

Table S2. G' and G'' values of the hydrogels in the presence of Mg^{2+} ions and 450 mM NaCl Page 8

Table S3. Assignment of the ATR vibration bands of chitin hydrogels Page 9

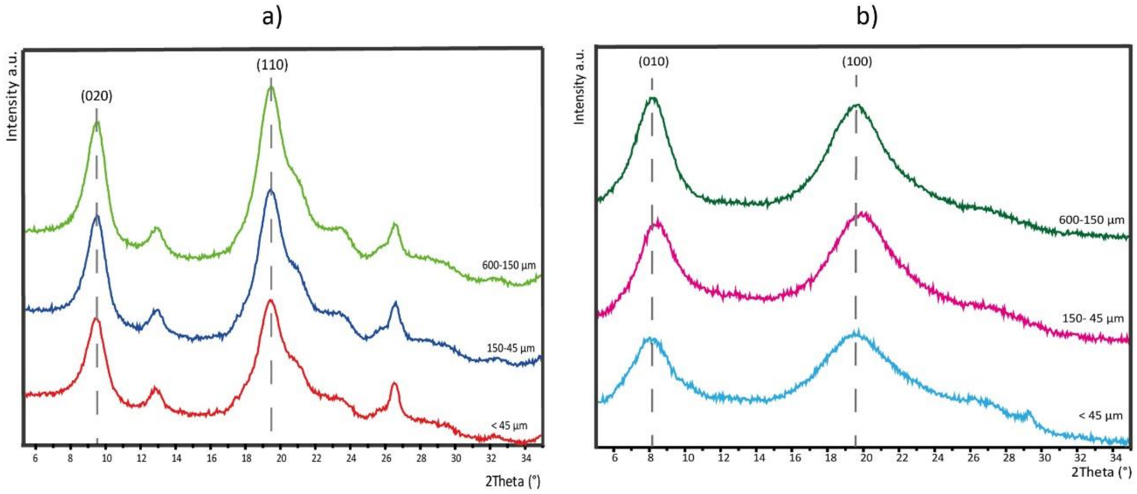

Figure S1.XRPD diffractogram of a) α-chitin powder sieved at various sizes. Peak (110) is the convolution of two peaks, as evidenced by the asymmetric profile. A decrease in crystallinity is observe decreasing the grain size and of b) β-chitin powder sieved at various sizes. A decrease in crystallinity is observe decreasing the grain size.

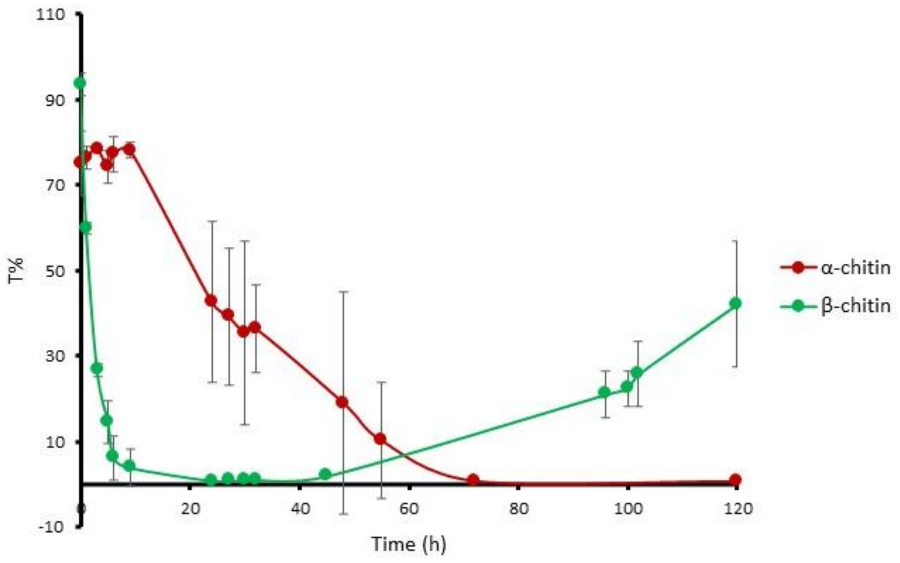

Figure S2. Transmittance variation during the oxidation of α-chitin and β-chitin.

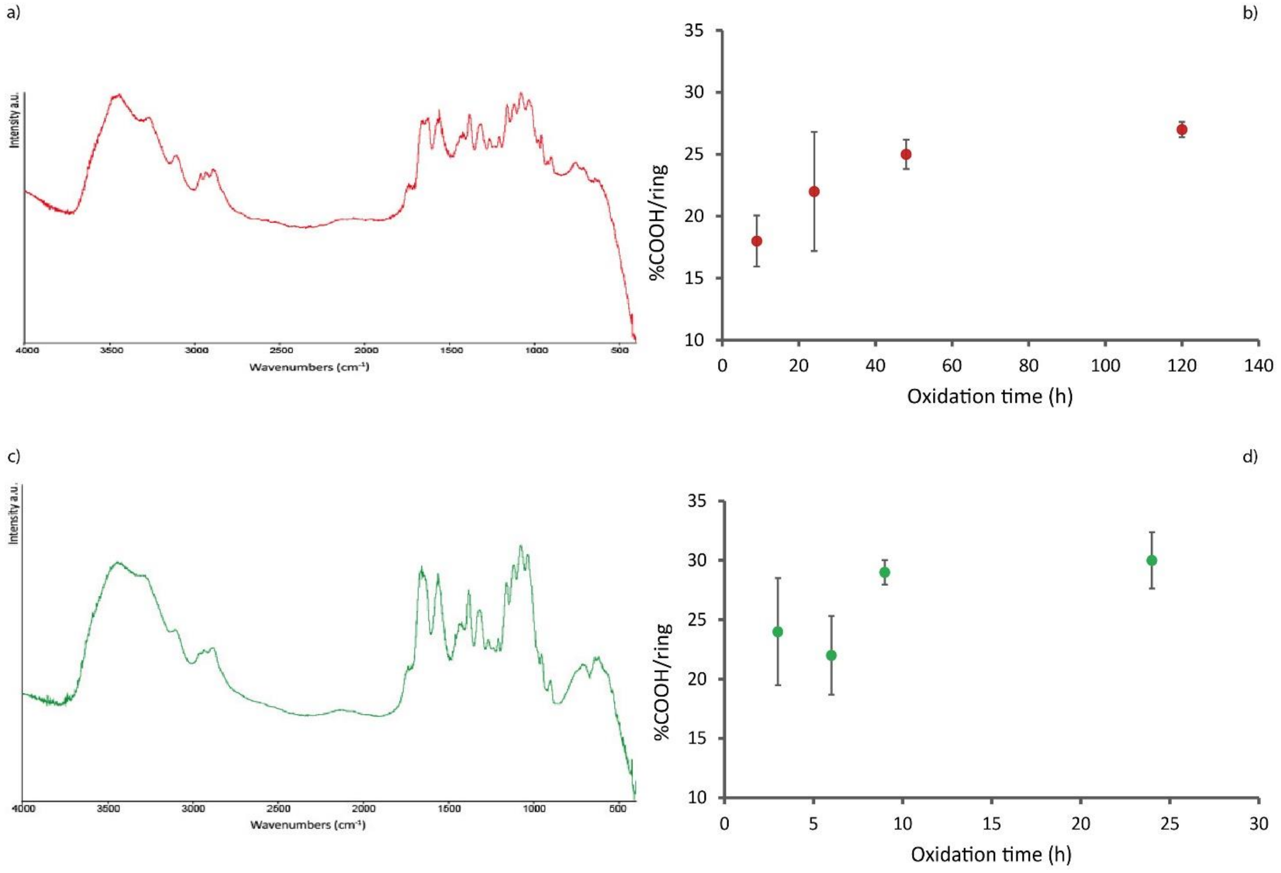

Figure S3. FTIR spectra of a) α-chitin oxidized for 120 hours and b) β-chitin oxidized for 24 hours The graphs show the relative intensity of the COOH band (1730 cm^{-1}) as the oxidation time of b) α and d) β-chitin increases.

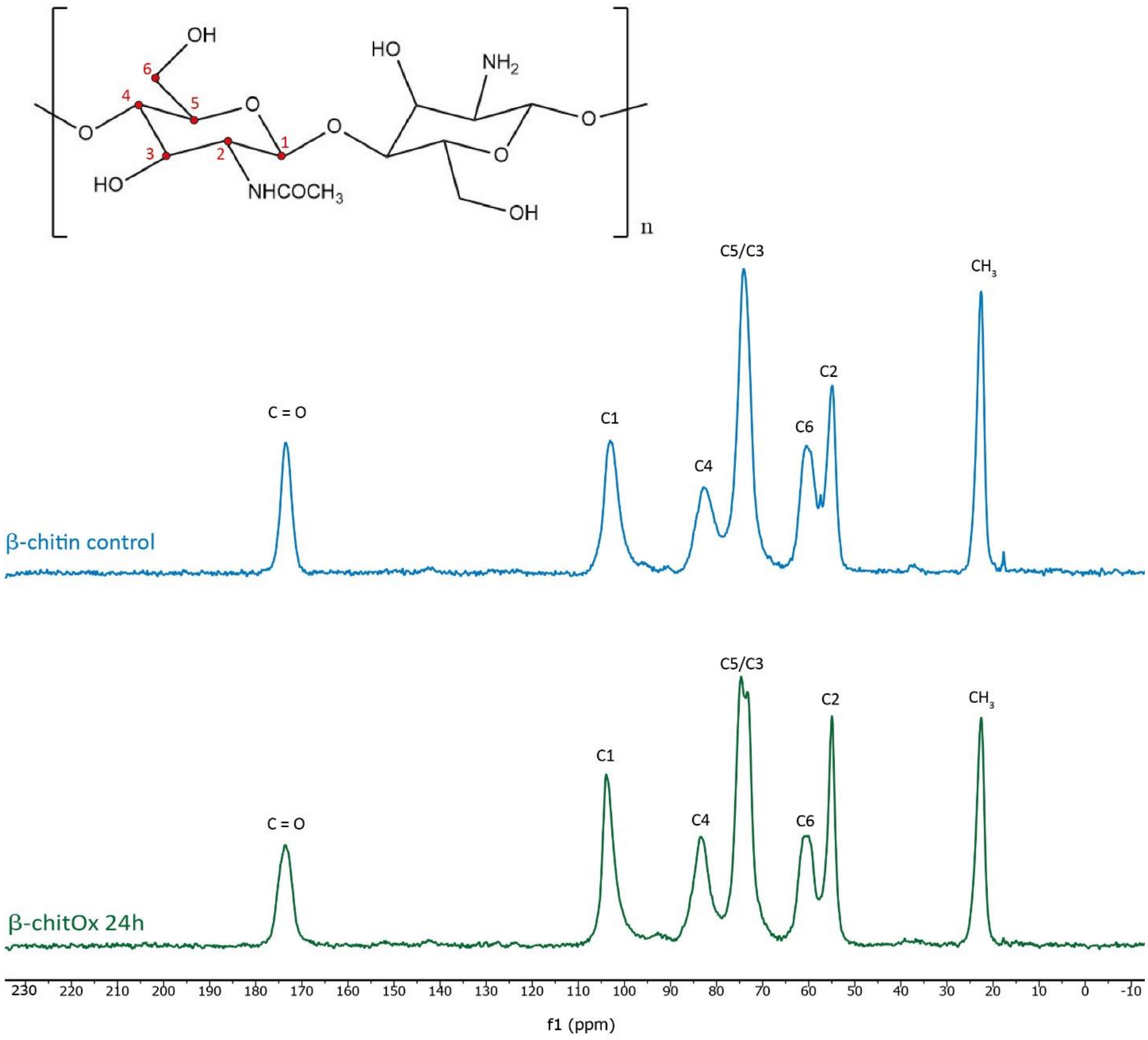

Figure S4. ss-NMR spectra of β-chitin control and oxidized β-chitin. The peaks obtained were assigned as shown in the image above [54,57]. In the spectrum of β-chitin oxidized for 24 hours, a decrease in the relative intensity of the C6 peak is observed.

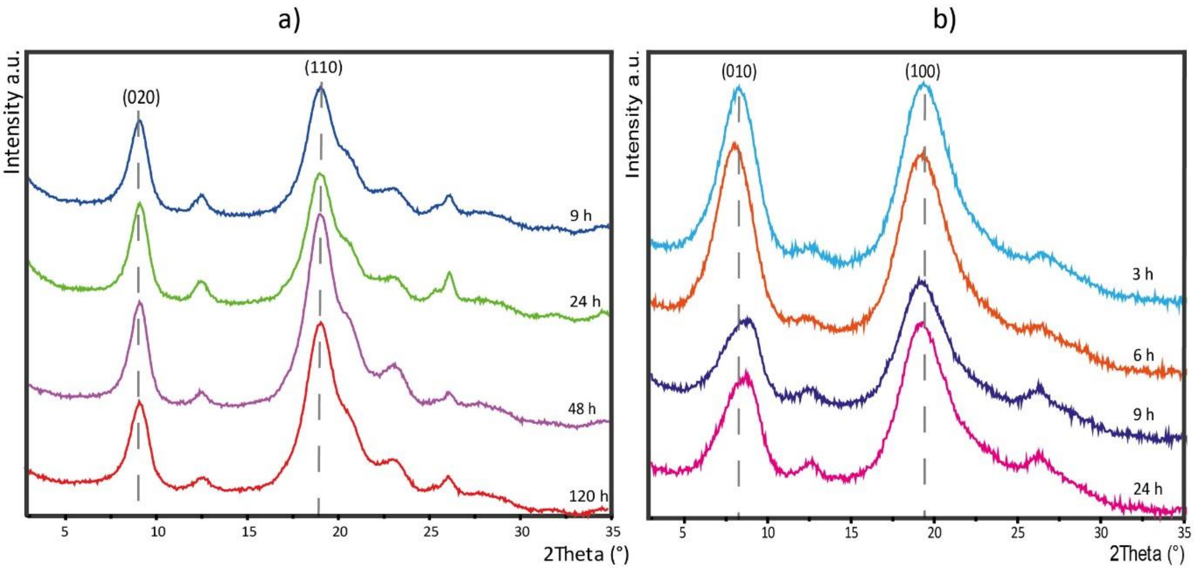

Figure S5. XRPD diffractogram of the different reaction products obtained from the oxidation of a) α-chitin at various times; b) β-*chitin at various times. Along the (010) direction a reduction of the cell parameter is observed increasing the reaction time. Conversely, along the (100) direction the crystallinity and the dimension of the lattice parameter increased.*

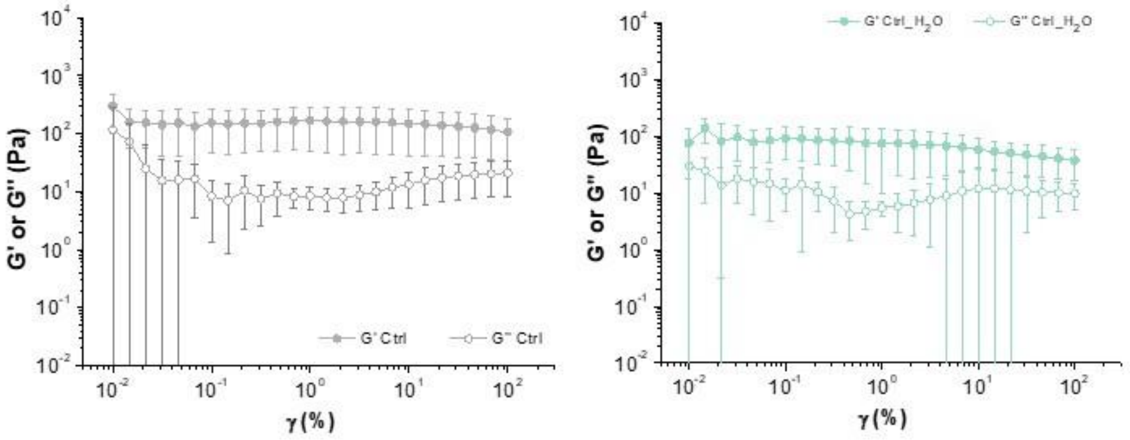

Figure S6. Amplitude sweep test of the ctrl and ctrl_H_2Osample. The bars indicate the error on the measurement.

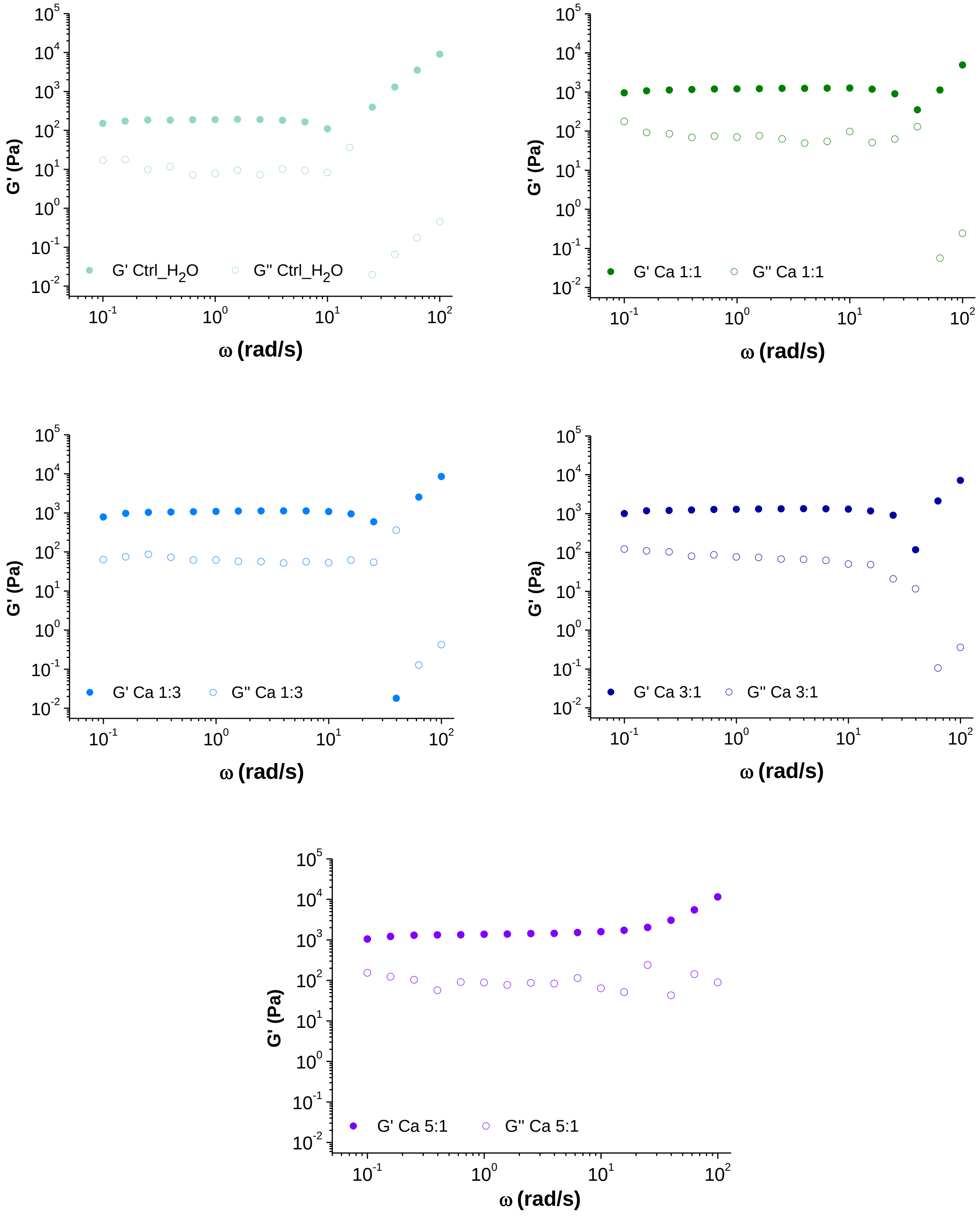

Figure S7. Frequency sweep of the samples: Ctrl_H_2O; Ca 1:1; Ca 1:3; Ca 3:1; Ca 5:1.

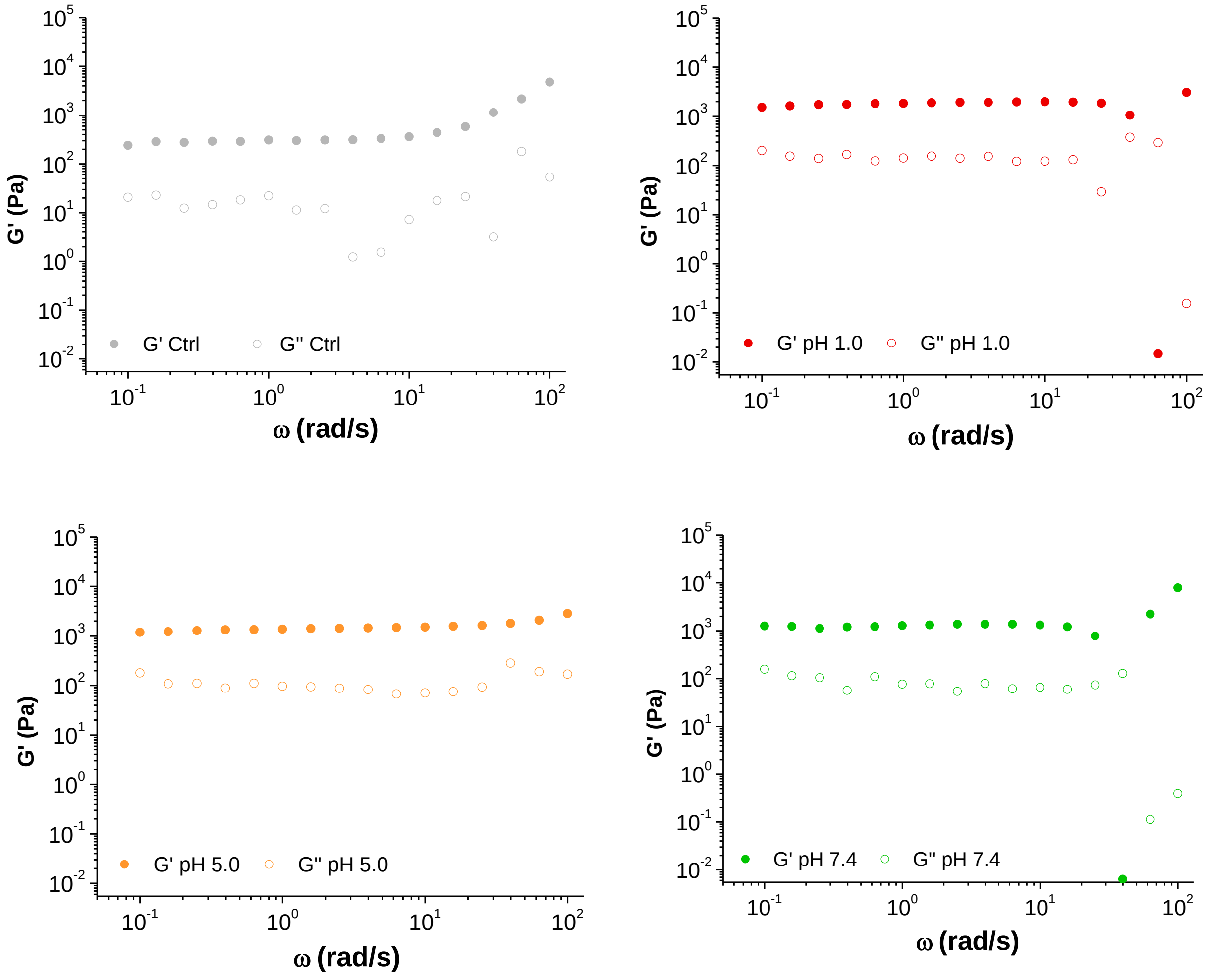

Figure S8. Frequency sweep of the samples: Ctrl; pH 1.0; pH 5.0; pH 7.4.

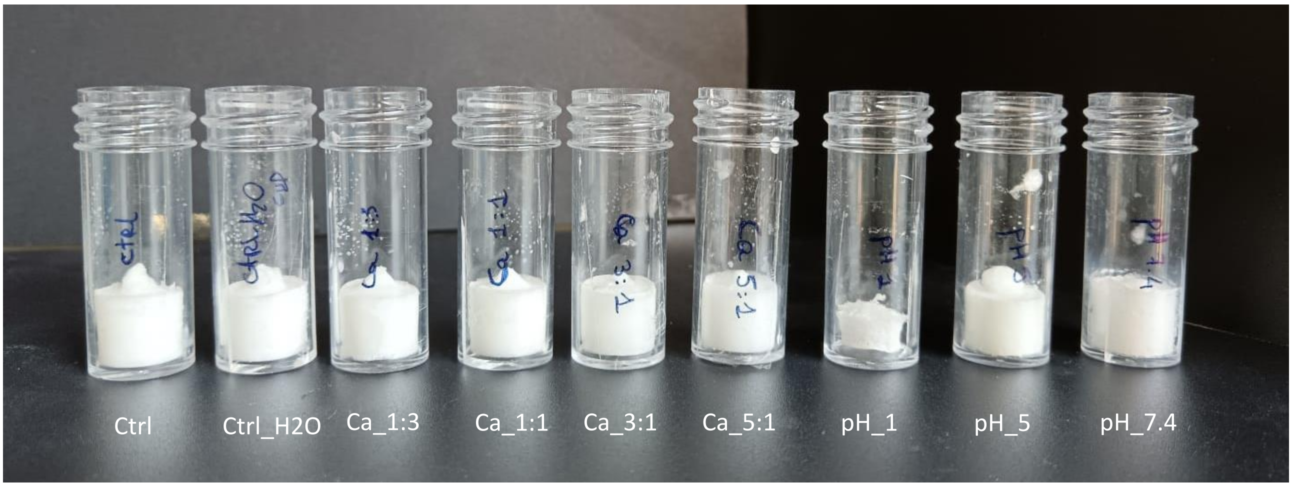

Figure S9. A picture reporting the hydrogels exposed to the different environments after freeze-drying. The image shows how the hydrogel maintained their original shape except for pH_1, which shows a shrinkage.

Table S1. G' and G'' values of the various addition methods tested corresponding to a shear strain of ɣ=0.046%. Sup= surface addition; Mix=mixed addition; Presonic = addition before sonication step.

Sample	Storage Modulus G'; (ɣ= 0,046%)	Loss Modulus G''; (ɣ= 0,046%)
	[Pa]	[Pa]
Ctrl	200 ± 100	20 ± 20
Ctrl_H_2Osup	80 ± 50	20 ± 10
Ctrl_H_2Omix	10 ± 20	20 ± 20
Ctrl_H_2O_presonic	100 ± 80	10 ± 10
Ca_1:1_mix	500 ± 100	60 ± 10
Ca_1:1_sup	690 ± 30	40 ± 20
Ca_1:1_presonic	120 ± 50	30 ± 20

Table S2. G' and G'' values of the hydrogels in the presence of Mg^{2+} ions and 450 mM NaCl. The values correspond to the shear strain of ɣ=0.046%.

Sample	Storage Modulus G'; (ɣ= 0,046%)	Loss Modulus G''; (ɣ= 0,046%)
	[Pa]	[Pa]
Ctrl	200 ± 100	20 ± 20
Ctrl_H_2Osup	80 ± 50	20 ± 10
Mg_3:1	800 ± 200	50 ± 10
NaCl	650 ± 70	10 ± 10

Table S3. Assignment of the ATR vibration bands of chitin hydrogels [52].

Vibrations mode	Ctrl_H_2O	Ca_1:3	Ca_1:1	Ca_3:1	Ca_5:1	Ctrl	pH 1.0	pH 5.0	pH 7.4
OH out of plane bending	691	689	684	689	667	688	669	689	686
Ring stretching	896	897	900	897	899	894	898	898	901
CH_3 wagging	951	951	952	952	952	952	949	951	950
C-O stretching	1029	1028	1027	1027	1025	1029	1026	1028	1028
C-O stretching	1068	1066	1065	1067	1064	1068	1060	1065	1063
Asymmetric in phase ring stretching mode	1111	1112	1111	1112	1110	1112	1108	1111	1111
Asymmetric bridge oxygen stretching	1154	1155	1154	1155	1153	1155	1153	1154	1153
Amide III	1203	1203	1202	1202	1203	1202	1203	1202	1202
band and CH_2 wagging	1308	1309	1309	1310	1312	1308	1309	1309	1310
CH bending and symmetric CH_3 deformation	1375	1375	1375	1377	1377	1375	1374	1375	1377
CH_2 bending and CH_3 deformation	1428	1430	1429	1429	1423	1429	1419	1420	1418
Amide II band	1555	1559	1554	1557	1557	1558	1559	1555	1557
Amide I band	1630	1636	1624	1627	1634	1634	1635	1624	1626
Amide I band	1655	1654	1653	1652	1651	1652	/	1654	1656
Symmetric CH_3 stretching and asymmetric CH_2 stretching	2876	2873	2874	2876	2880	2873	2874	2874	2874
CH_3 stretching	2920	2935	2935	2937	2935	2930	2930	2935	2937
N-H stretching	3099	3100	3101	3100	3108	3094	3090	3100	3108
N-H stretching	3281	3272	3263	3271	3256	3280	3255	3275	3263
O-H stretching	3435	3427	3448	3425	3372	3437	/	3421	3394

n.b. All reported values are expressed in cm^{-1}.

References

[52] Montroni, D.; Fermani, S.; Morellato, K.; Torri, G.; Naggi, A.; Cristofolini, L.; Falini, G. β-Chitin Samples with Similar Microfibril Arrangement Change Mechanical Properties Varying the Degree of Acetylation. *Carbohydr. Polym.* **2019**, *207*, 26–33. https://doi.org/10.1016/j.carbpol.2018.11.069.

[54] Montroni, D.; Sparla, F.; Fermani, S.; Falini, G. Influence of Proteins on Mechanical Properties of a Natural Chitin-Protein Composite. *Acta Biomater.* **2021**, *120*, 81–90. https://doi.org/10.1016/j.actbio.2020.04.039.

[57] Wang, Q.; Yan, X.; Chang, Y.; Ren, L.; Zhou, J. Fabrication and Characterization of Chitin Nanofibers through Esterification and Ultrasound Treatment. *Carbohydr. Polym.* **2018**, *180*, 81–87. https://doi.org/10.1016/j.carbpol.2017.09.010.
